# Major dietary patterns in relation to muscle strength status among middle‐aged people: A cross‐sectional study within the RaNCD cohort

**DOI:** 10.1002/fsn3.2617

**Published:** 2021-10-10

**Authors:** Mehnoosh Samadi, Tina Khosravy, Leila Azadbakht, Mansour Rezaei, Mohammad Mosafaghadir, Negin Kamari, Amir Bagheri, Yahya Pasdar, Farid Najafi, Behrouz Hamze, Davood Soleimani

**Affiliations:** ^1^ Department of Nutritional Sciences School of Nutritional Sciences and Food Technology Kermanshah University of Medical Sciences Kermanshah Iran; ^2^ Department of Nutritional Sciences Research Center for Environmental Determinants of Health (RCEDH) Health Institute Kermanshah University of Medical Sciences Kermanshah Iran; ^3^ Department of Health and Nutrition Lorestan University of Medical Science Lorestan Iran; ^4^ Department of Community Nutrition School of Nutritional Sciences and Dietetics Tehran University of Medical Sciences Tehran Iran; ^5^ Biostatistics and Epidemiology Department School of Public Health Kermanshah University of Medical Sciences Kermanshah Iran; ^6^ Department of Epidemiology Research Center for Environmental Determinants of Health (RCEDH) Health institute Kermanshah University of Medical Sciences Kermanshah Iran; ^7^ Department of Public Health Kermanshah University of Medical Sciences Kermanshah Iran; ^8^ Research Center of Oils and Fats Kermanshah University of Medical Sciences Kermanshah Iran

**Keywords:** dietary pattern, grip strength, middle‐aged adults, muscle strength

## Abstract

Grip strength in midlife can predict physical disability in senior years. Recent evidence shows the critical role of nutritional status on muscle function. We aimed to elucidate whether adherence to a particular dietary pattern would be associated with abnormal muscle strength among middle‐aged people. In this cross‐sectional study, a semiquantitative Food Frequency Questionnaire was used to assess the dietary intake of 2781 participants in the Ravansar Non‐Communicable Chronic Disease (RaNCD) cohort. Major dietary patterns from 28 main food groups were extracted using principal component analysis. Binary logistic regression was used to determine the association between the tertiles of the major dietary patterns and muscle strength status. Two major dietary patterns were identified: the “mixed dietary pattern” that heavily loaded with fruits, vegetables, nuts, dairies, sweets, legumes, dried fruits, fish, red meat, butter, whole grains, natural juices, poultry, pickles, olive, industrial juice, egg, processed meat, and snacks and “unhealthy dietary pattern” that heavily loaded by fats, sugar, refined grains, soft drink, salt, organ meat, tea, and coffee. Adherence to the mixed dietary pattern (OR = 1.03, 95% CI = 0.8–1.33, P for trend = 0.77) and the unhealthy dietary pattern (OR = 1.01, 95% CI = 0.79–0.13, P for trend = 0.89) did not associate with abnormal muscle strength. This study suggests that the dietary pattern involving the consumption of healthy and unhealthy food does not have an effect on muscle strength in middle‐aged adults.

## Graphical abstract

We identified two major dietary patterns from data of participants in the Ravansar Non‐Communicable Chronic Disease (RaNCD) study. The first dietary pattern was “mixed” that heavily loaded with fruits, vegetables, nuts, dairies, sweets, legumes, dried fruits, fish, red meat, butter, whole grains, natural juices, poultry, pickles, olive, industrial juice, egg, processed meat, and snacks and the second dietary pattern was “unhealthy” that heavily loaded by fats, sugar, refined grains, soft drink, salt, organ meat, tea, and coffee. Surprisingly we found that adherence to the mixed dietary pattern and the unhealthy dietary pattern did not associate with abnormal muscle strength.

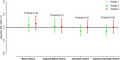

## 1 INTRODUCTION

Handgrip strength (HGS) is a good predictor of general health, nutritional status, physical disability, muscle loss, and morbidity (Gallup et al., 2007); thus, a strong HGS in midlife can indicate the capacity to prevent illness and reduce physical disability and aging‐related outcomes in future (Rantanen et al., 2000). HGS has shown prognostic utility in the assessment of aging‐related outcomes (Lawman et al., 2016). After 30, aging leads to an approximately 1% decline in muscle mass for each year (Kim & Choi, 2013). Aging after middle age causes a change in the body composition, which involves reducing muscle mass and increasing body fat mass. The main concern about aging is loss of muscle mass that leads to physical inability and mortality (Houston et al., 2008). Sarcopenia is a syndrome characterized by the progressive loss of the entire muscle and muscle strength with complications of physical inability, poor quality of life and death. The reasons for the onset and progression of sarcopenia are the inadequate food intake, inhibition of absorption, inactivity and endocrine disorders such as insulin resistance (Baumgartner et al., 1998; Cruz‐Jentoft et al., 2010; Morley et al., 2001).

Nutritional status is an important factor in body's health and its role as a determinative factor for chronic diseases has been proven (Organization, 2002). Nutrition plays an important role in maintaining muscle performance, muscle strength and decreasing rate of age‐related skeletal muscle mass loss, so that optimizing diet and nutrition throughout life can be a key for preventing sarcopenia and enhancing physical fitness (Robinson et al., 2012). Robert et al. in the Framingham Offspring Cohort study indicated that higher consumption of protein can play a protective role in reducing grip strength (McLean et al., 2015). Sian et al. showed that high consumption of fatty fish can improve grip strength in older men and women (Robinson et al., 2008). A prospective study revealed that vitamin D can play an important role in preserving muscle mass and performance (Scott et al., 2010). Richard et al. in their cross‐sectional study indicated that high levels of beta‐carotene and vitamin E can help increase muscle strength (Semba et al., 2003). The result of InCHIANTI study showed that high level concentration of magnesium influence on muscle performance such as grip strength in older adult (Dominguez et al., 2006a).

Since consumed foods are a combination of nutrients and nonnutrients, it is better to investigate the dietary patterns individually rather than nutrients alone for identification of diet‐disease relationships (Esmaillzadeh et al., 2007b). Some studies showed that healthy or prudent dietary patterns were positively associated with muscle strength (Lee, 2020; Zhang et al., 2020). However, other study indicated that subjects with unhealthy dietary patterns had a significantly lower muscle strength (Granic et al., 2016; Kang et al., 2020). Due to no studies conducted on Iranian population, we examined the association between adherence to each dietary pattern and odds of abnormal muscle strength.

## 2 MATERIALS AND METHODS

### 2.1 Study population

The current cross‐sectional study was carried on data from participants in Ravansar Non‐Communicable Chronic Disease (RaNCD) study. We use from following formula for estimation of the sample size in cross‐sectional study.

$$N=\frac{{\left(z_{1‐\frac{a}{2}}+z_{1‐B}\right)}^{2}\left(\sigma 1^{2}+\sigma 2^{2}\right)}{{\left(\mu_{1}‐\mu_{2}\right)}^{2}}=\frac{{\left(1.96+1.28\right)}^{2}\left(2.7^{2}+2.5^{2}\right)}{{\left(76.6‐73.9\right)}^{2}}=20$$

According to Hashemi, R study, *Z*_1‐α/2_ = 1.96 for 95% standard, *Z*_1‐_ *_B_* = 1.28, µ_1_ = 76.6 ± 2.7, µ_2_ = 73.9 ± 2.5. *N* = 20 is the minimum number of sample size for each dietary pattern, we estimated at least 3 dietary pattern to result in this population, since the current cross‐sectional study was carried on data from participants in Ravansar Non‐Communicable Chronic Disease (RaNCD) study, after adjusting for inclusion and exclusion criteria we used 2781 subjects for assessment of dietary intake and dietary pattern. RaNCD study was designed by a Persian cohort in the rural and urban areas of Ravansar in the west of Kermanshah province (Iran) in 2014 for identifying noncommunicable chronic diseases. In the RaNCD cohort study, 10,065 males and females within the age range of 35–65 years were evaluated. Subjects from the city of Ravansar were voluntarily enrolled in this study after referring to Ravansar Health Center. In this study, 4600 subjects did not have muscle strength information and 2684 subjects with diabetes mellitus, chronic renal failure, endocrine disorders, liver diseases, inflammatory diseases, professional athletes, use tobacco, alcohol, dietary supplements, energy intake outside the normal range (800–4200 kcal/day), and taking steroid anti‐inflammatory drugs were excluded from our investigation, and 2781 subjects were examined. Ethical approval was given by the Ethics Committee of the Deputy of Research and Technology of Kermanshah University of Medical Sciences, Kermanshah, Iran (Ethic number: IR.KUMS.REC.1396.512).

### 2.2 Body composition analysis

Body composition of all subjects was determined using multifrequency Bio Impedance Analysis (BIA, InBody 770, Biospace Ltd, Seoul, Korea). The height was measured by BSM 370 (Biospace Co, Seoul, Korea) to the nearest 0.1 cm. The body mass index (BMI) was calculated by dividing weight in kilograms by the height in squared meters (kg/m^2^). Obesity and overweightness were evaluated based on WHO standard criteria as follows: BMI ranges from 25 to 29.9 kg/m^2^ is overweight and Obesity is BMI equal to or greater than 30 kg/m^2^ (Organization, 2006).

### 2.3 Assessment of dietary intake

A semiquantitative 125‐item Food Frequency Questionnaire (FFQ) was used to assess dietary intake over a year. The FFQ includes a list of food with standard service sizes consumed by Iranian population (Esmaillzadeh et al., 2007c; Mirmiran et al., 2009). The reported frequency for each food item was then converted to a daily intake. Portion sizes of consumed foods were converted to grams using household measures (Lopez‐Garcia et al., 2004). The energy, micronutrients and macronutrients content of food were obtained by Nutritionist IV (N4) software (version 7.0; N‐Squared Computing, Salem, OR, USA) based on United states Department of Agriculture (USDA) food composition table revised for Iranian foods (Azar & Sarkisian, 1980). Dietary intake was excluded in the final analysis if the energy intake was not within the normal range (800–4200 kcal/day).

### 2.4 Assessment of physical activity

Physical activity levels were evaluated by the International Physical Activity Questionnaire (IPAQ), which included questions about intense, moderate and low‐level physical activity in the 7‐day period. The reliability and validity of this questionnaire was confirmed previously in the Iranian population (Vasheghani‐Farahani et al., 2011). According to the IPAQ, each person's physical activity is reported based on the metabolic equivalent (MET h/week) (Committee, 2005), each all of the questionnaires were completed by trained dietitians in face‐to‐face interview. Physical activity lower than 7.5 MET hours per week equals to low, physical activity ≥7.5 MET hours per week <21, equals to normal or moderate, and physical activity ≥21 MET hours per week equals to high physical activity.

### 2.5 Assessment of muscle strength

The muscle strength was measured by the handgrip test with the use of a digital dynamometer (model SH5003, Seahan Co, South Korea). The handgrip strength was taken with right/dominant hand when the participant was sitting and the elbow was at 90° of flexion. The participants were asked to squeeze the handle with maximal effort for 10 s. The measurement was repeated after 30 s and the latter was recorded as hand grip strength. We used the cut‐off points for muscle strength suggested in Lauretani study (Lauretani et al., 2003) in which muscle strength lower than 20 kg for women and lower than 30 kg for men considered abnormal muscle strength.

### 2.6 Statistical analysis

All data were analyzed with the use of the SPSS software version 16 (SPSS Inc., Chicago, IL, USA). Factor analysis was used to identify dietary patterns. Food items in the FFQ were categorized into 28 main food groups based on the similarity of nutrient profiles or culinary usage (Table 1). We used the Kaiser–Meyer–Olkin (KMO) and Bartlett's sphericity test to evaluate the adequacy of the intercorrelation between food groups for the factor analysis. The number of retained dietary patterns was decided by the Eigenvalue of more than 1 and the shape of the scree plot and then Varimax rotation was run to create a simple and distinct matrix for preferable explanation and uncorrelated dietary patterns. The scores of each dietary pattern was determined by summing the intake of food groups weighted by factor loading of these food groups for each pattern. The main components of each dietary pattern were determined according to the rotated factor loading (absolute) more than 0.2 (Table 2). The positive factor loading in each pattern indicates a direct relation with pattern and a negative factor loading indicates an inverse relation with pattern.

**TABLE 1:** Food grouping used in the dietary pattern analyses

Food groups	Food items
Processed meats	Hamburgers, sausages
Red meat	Beef and veal, sheep, minced meat
Organ meat	Heart, liver, tripe, kidney, brain, tongue
Fish	Canned tuna fish, other fish.
Poultry	Chicken
Egg	Egg
Low‐fat dairy products	Low‐fat milk, Low‐fat yogurt, doogh (A drink made from a mixture of water and yogurt)
High fat dairy products	High fat milk, chocolate milk, high fat yogurt, cream, ice cream, cheese
Tea, coffee	Tea, coffee
Nut	Peanut, almonds, pistachios, hazelnuts, walnuts, roasted seeds
Legumes	Lentils, beans, chick peas, split peas, Soya and other legumes
Fruit	Apricots, cantaloupes, tangerine, plums, cherries, oranges, persimmons, peaches, Pears, apples, grapes, bananas, watermelon, kiwi, strawberries, mulberry, pomegranates, lemons, figs, dates and greengages.
Natural juices	Natural water fruits
Commercial fruit juice	Industrial Juice
Vegetables	Lettuce, winter squash, green peas, carrot, cabbage, sweet peppers, spinach, turnip, vegetables (basil), Corn, broad beans, cucumber, cooked vegetables, zucchini, eggplant, celery, green beans, garlic, onion, mushroom, Tomatoes
Whole grains	Dark breads (Iranian), barely, bulgur, corn
Refined grain	White breads (lavash, baguettes, taftun, barbary), pasta, rice, biscuits
Dried fruit	Raisins, currant, Dried fruits such as fig, Berries, Apricot, Peach
Olive	Olive, olive oil
Snacks	French fries, potato chips, cheese puffs
Sweets	Chocolates, cookies, cakes, confections
Saturated fats	Solid oil, animal fats
Butter	Butters, Margarine, Mayonnaise
Unsaturated fats	Vegetable oils (except olive oil)
Salt	Salt
Soft drinks	Soft drinks
Pickles	Pickles
Sugar	Sugar

**TABLE 2:** Factor‐loading matrix for major dietary patterns

Food groups	Mixed diet	Unhealthy diet
Fruit	0.639	‐
Vegetables	0.586	‐
Nuts	0.476	‐
High fat dairy products	0.475	‐
Sweets	0.47	0.242
Legumes	0.448	‐
Dried fruits	0.421	‐
Fish	0.405	‐
Red meat	0.404	‐
Butter	0.399	0.244
Whole grain	0.374	‐
Natural juice	0.357	‐
Poultry	0.351	‐
Pickles	0.338	‐
Olive	0.305	−0.26
Commercial fruit juice	0.303	0.24
Egg	0.291	0.272
Processed meats	0.291	‐
Low‐fat dairy products	0.283	‐
Snack	0.281	0.269
Saturated fats	‐	0.568
Sugar	‐	0.553
Unsaturated fats	0.298	−0.392
Refined grain	‐	0.383
Soft drink	0.318	0.368
Salt	‐	0.367
Organ meat	0.301	0.365
Tea, coffee	‐	0.313
Percentages of variance explained (%)	12.85	7.06

Note

Absolute values less than 0.2 are not displayed for simplicity.

To assess the normality assumption, the Kolmogorov‐Smirnov test was used. We used the one‐way analysis of variance (ANOVA) to assess the significance of difference for continuous variable and Chi‐squared test for categorical variables in tertile categories of the dietary pattern. Binary logistic regression in the two models was run to estimate the odds ratio (OR) for abnormal muscle strength to follow each dietary pattern. The first model was crude and the second model (model I) was adjusted for confounding factors such as age, gender, physical activity, educational level, economical level, and energy intake. Finally, the Hosmer‐Lemsho test was performed on the data. A two‐sided *p* value of less than .05 was considered to indicate statistical significance.

## 3 RESULTS

The present study included data from 2781 healthy men and women. Demographic characteristics, anthropometric parameters and muscle strength of the participants are shown in Table 3. Using factor analysis, two major dietary patterns were identified: “mixed dietary pattern” and “unhealthy dietary pattern.” Mixed dietary pattern was characterized by high intake of fruits, vegetables, nuts, dairy, sweets, legumes, dried fruits, fish, red meat, butter, whole grains, natural juices, poultry, pickles, olive, industrial juice, egg, processed meat, and snacks. Unhealthy dietary pattern was characterized by high intake of fat, sugar, refined grains, soft drink, salt, organ meat, tea, and coffee and low consumption of oil. These two dietary patterns explained 19.91% of the total variation in our sample.

**TABLE 3:** General characteristics and dietary intakes of study participants

Variables	Value
Age, years	8 ± 47.3
Female, *n* %	64.2
Weight, kg	13 ± 70.2
Body mass index, kg/m^2^	4.5 ± 27.1
Muscle mass, kg	1.1 ± 9.5
Fat mass, kg	3.9 ± 9.7
Physical activity, MET h/week	6.8 ± 40.9
Hand grip strength, kg	
Men	9.2 ± 40.77
Women	5.6 ± 23.92
Education *n* %	29.4
Illiterate	
Under the diploma	53.7
Diploma	11.4
Super‐diploma and higher	5.5
Dietary intake	
Total energy, kcal/day	658 ± 2,792
Carbohydrate*, g/day	105.1 ± 423.2
Protein*, g/day	28.6 ± 93.3
Fat*, g/day	25.6 ± 83.9
Abnormal muscle strength cases, *n*	549

Note

Muscle strength lower than 20 kg for women and lower than 30 kg for men considered abnormal muscle strength. Data are reported as mean ± standard deviation or percentage as appropriate.

* Adjusted for total energy intake.

**FIGURE 1:**
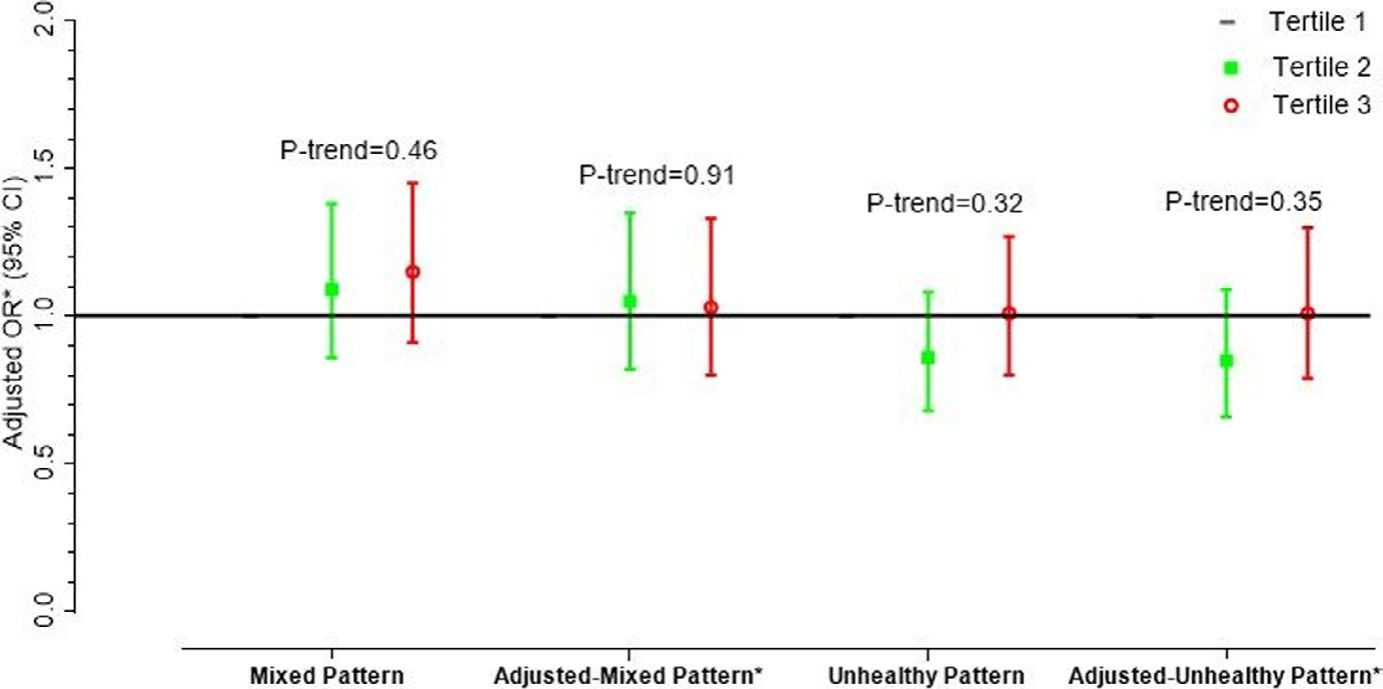
Odds ratios (95% CI)* for abnormal muscle strength across tertiles (T) of dietary pattern scores. Odds ratios (95% CI) were obtained using binary logistic regression. *Adjusted for sex, age, education level, economical status, physical activity, and energy intake. Hosmer‐Lemeshow Χ^2^ (8) = 11.70. Prob > Χ^2^ = 0.1650

The general characteristics of participants in tertile categories of the major dietary pattern are shown in Table 4. In the mixed dietary pattern, intake of energy, protein and fat in the highest tertile (T3) was lower than first tertile (T1) and in the unhealthy dietary pattern, the fat intake of the participants in the highest tertile (T3) was significantly higher than the first tertile (T1). Table 5 shows the anthropometric measurements, muscle strength, and relative frequency of abnormal muscle strength in the tertile categories of the major dietary patterns. In the mixed dietary pattern, mean weight, BMI, and muscle mass of the participants in highest tertile (T3) were lower than first tertile (T1). There was no significant difference in abnormal muscle strength in tertile categories of the two major dietary pattern. OR and 95% CI for risk of abnormal muscle strength in tertile categories of the major dietary pattern are shown in Figure 1. The first tertile of each dietary pattern was considered as reference category. In the crude model, we did not observe any significant association between the major dietary pattern and abnormal muscle strength. These associations remained without change after adjusting the age, sex, educational level, economical status, total energy intake, and physical activity levels (Adjusted Model). On the other hand, the Hosmer‐Lemsho test results were not significant (*p* = .051).

**TABLE 4:** General characteristics and dietary intakes of study participants in tertiles (T) of dietary pattern

Variables	Mixed dietary pattern				Unhealthy dietary pattern			
	T1	T2	T3	*p*	T1	T2	T2	*p*
	(*N* = 927)	(*N* = 919)	(*N* = 925)		(*N* = 930)	(*N* = 945)	(*N* = 945)	
Age, years	47.1 ± 8	47.2 ± 8	47.7 ± 8.2	.18	47.2 ± 8.2	47.2 ± 8	47.6 ± 8	.4
Female, %	62.5	65	64.7	.47	0.64	65.5	62.7	.46
PA, MET h/w	41 ± 7	40.7 ± 6.6	40.9 ± 6.7	.53	40.7 ± 6.6	41.1 ± 6.9	40.9 ± 6.8	.46
Energy, kcal/day	2841 ± 639	2760 ± 660	2773 ± 673	.01	2756 ± 661	2794 ± 652	2826 ± 661	.07
Carbohydrate, g/day	429.2 ± 102.8	418.1 ± 105	422.1 ± 107.2	.07	419 ± 104.9	424 ± 104.7	426.5 ± 105.8	.29
Protein, g/day	95.3 ± 27.8	92.6 ± 28.6	92 ± 29.4	.02	91.9 ± 28.8	93.7 ± 28.4	94.3 ± 28.6	.15
Fat, g/day	86.1 ± 25	82.9 ± 25.2	82.6 ± 26.5	.001	82.2 ± 25.2	83.6 ± 24.6	85.9 ± 26.9	.001

Note

Data are reported as mean ± standard deviation or percentage as appropriate. *p* Values were calculated using ANOVA test for quantitative variables and chi‐square test for qualitative variables.

Abbreviations: MET, Metabolic Equivalent; PA, Physical Activity.

**TABLE 5:** Anthropometric measurements, muscle strength and the prevalence of abnormal muscle strength in different tertiles of dietary pattern scores

Variables	Unhealthy dietary pattern				Mixed dietary pattern			
	T1	T2	T3	*p*	T1	T2	T3	*p*
	(*N* = 927)	(*N* = 919)	(*N* = 925)		(*N* = 930)	(*N* = 945)	(*N* = 896)	
Weight, kg	13.4 ± 71.3	12.7 ± 69.6	12.9 ± 69.7	.01	12.2 ± 69.5	13.5 ± 70.8	13.2 ± 70.3	.07
Body mass index, kg/m^2^	4.7 ± 27.4	4.5 ± 27	4.4 ± 26.9	.02	4.4 ± 26.9	4.6 ± 27.2	4.6 ± 27.1	.35
Muscle mass, kg	1.1 ± 9.6	1.1 ± 9.4	1.1 ± 9.5	.01	1.1 ± 9.5	1.2 ± 9.6	1.2 ± 9.5	.08
Fat mass, kg	4 ± 9.9	3.9 ± 9.7	3.8 ± 9.5	.19	3.8 ± 9.6	3.9 ± 9.8	4 ± 9.7	.79
Hand grip strength, kg	10.4 ± 30.3	10.1 ± 29.5	10.6 ± 30.1	.21	10.2 ± 29.8	10.5 ± 30.2	10.3 ± 30	.78
Abnormal muscle strength, *n*%	18.3	19.9	20.9	.35	20.5	17.9	20.7	.24

Note

Muscle strength lower than 20 kg for women and lower than 30 kg for men considered abnormal muscle strength. Data are reported as mean ± standard deviation or percentage as appropriate. *p* Values were calculated using ANOVA test for quantitative variables and chi‐square test for qualitative variables.

## 4 DISCUSSION

This study, to the best of our knowledge, is the first study to assess the relation between adherence to the certain dietary patterns and muscle strength among middle‐aged men and women. Previous studies focused on the association between dietary pattern and the risk of sarcopenia and the weakness in older adults or exclusively on association between one macronutrient or micronutrient intake and muscle function in elderly or middle‐aged individuals (Chan et al., 2016; Dominguez et al., 2006b; Fanelli Kuczmarski et al., 2013; Hashemi et al., 2015; Kim et al., 2013a, 2013b; Paddon‐Jones et al., 2008; Robinson et al., 2008; Scott et al., 2010). We identified two major dietary patterns in our sample using factor analysis: “mixed dietary pattern” and “unhealthy dietary pattern.” The two dietary patterns were identified in this study are different compared to other studies conducted in Iran (Hashemi et al., 2015; Mohseni et al., 2017). These differences may be due to different dietary habits in cultures in Iran. Our findings did not show the significant association between major dietary patterns and abnormal muscle strength.

Based on the previous studies, inflammation is associated with muscle wasting and sarcopenia (Allen, 2017; Jensen, 2008; Schaap et al., 2006). Inflammation causes an imbalance between protein synthesis and catabolism (Jo et al., 2012). In this study, the mixed dietary pattern included a collection of healthy foods such as fruits, vegetables, nuts, olive, fish, natural juices, and whole grain and unhealthy foods such as red meat, processed meat, snacks, butter, high fat dairy product, and pickles. Other studies described the role of healthy foods in improving muscle strength and performance by reducing inflammation (Berendsen et al., 2013). Robinson et al. reported that fish and nut consumption can improve muscle function in older adult by possible anti‐inflammatory role of n‐3 fatty acid (Robinson et al., 2008). A prospective study reported that nut consumption was associated with lower risk of physical dysfunction (Arias‐Fernández et al., 2018). Pierno et al., (2014) reported that olive oil derived antioxidant can reduce skeletal muscle function loss during aging. Endothelial dysfunction may contribute to the development of sarcopenia (Timmerman & Volpi, 2013). Fish, nut and olive oil consumption can be associated with decreased endothelial dysfunction (van Bussel et al., 2011; Cortés et al., 2006; Kasliwal et al., 2015; Palmieri et al., 2012). The possible reason for this effect can be due to modified membrane stability and fluidity, endothelial nitric oxide activity, decreased inflammation and prevention of intracellular inflammatory signaling pathways by fish, nut, and olive oil consumption (van Bussel et al., 2011; Ma et al., 2010; Perona et al., 2006). Millward indicated that the intake of fruits and vegetable that are a rich source of antioxidants can reduce inflammation and prevent sarcopenia (Millward, 2012). Also, in line with these findings, it was reported that 5 serving/day fruits and vegetables consumption compared with 2 serving/day leads to increased grip strength in older adults (Wu et al., 2004). In a cross‐sectional study, high fruit and vegetable intake was inversely associated with sarcopenia in older adults (Kim et al., 2015). Also, in the Hertfordshire cohort study, higher fruit consumption in older men and higher fruit and vegetable consumption in older women was associated with higher grip strength (Robinson et al., 2008). Fruits and vegetable are sources of antioxidants such as vitamin C and carotenoids (Wu et al., 2004). These compounds can prevent muscle catabolism by reducing oxidative stress (Doria et al., 2012). Carotenoids play a protective role in keeping the muscle strength by scavenging free radicals, suppressing singlet oxygen and preventing lipid peroxidation (Young et al., 2004). Vitamin C can play a protective role in muscle strength both directly by interacting with superoxide and hydroxyl‐free radical (Rose & Bode, 1993), and indirectly by reducing vitamin E radicals (Chan, 1993). Several studies indicated that high whole grain intake has been accompanied with reduced inflammation and oxidative stress (Hoffmann et al., 2004; Jang et al., 2001; Schulze et al., 2005). Whole grains contain compounds that can play an antioxidant role and reduce inflammation damage through mechanisms such as cell cycle control, protein chaperoning and repair, DNA stabilization, and elimination of reactive molecular species and induction of detoxification mechanisms (Blomhoff, 2005; Gutteridge & Halliwell, 2000). On the other hand, unhealthy foods can lead to increased inflammation (Chan, 1993). Esmaillzadeh et al. reported that the western dietary pattern characterized with high consumption of unhealthy foods is associated with inflammatory factors (Esmaillzadeh et al., 2007a). Another study by Garcia at al. indicated that there was a positive relation between western dietary pattern and inflammation (Lopez‐Garcia et al., 2004). Due to high content of fat, especially saturated fatty acids in red and processed meat, their high consumption could be associated with inflammation (Meyer et al., 2011). A dietary pattern with high loading fats, oils, processed food, fried potatoes, salty snacks, and deserts play a role in inflammation etiology (Nettleton et al., 2006). Therefore, our findings might be attributable to neutralizing the anti‐inflammatory effects of healthy foods by the inflammatory effects of unhealthy foods in the mixed dietary pattern.

We also did not find any association between the unhealthy dietary pattern and abnormal muscle strength. This finding is unexpected because this dietary pattern included higher level of consumption of saturated fats, sugars, refined grains, soft drink, salt, and organ meat. Several studies reported that consumption of saturated fat could induce inflammation (Fung et al., 2001; Kennedy et al., 2009; King et al., 2003). Masters et al. reported that refined grain consumption is associated with inflammation (Budui et al., 2015; Masters et al., 2010). Dietary pattern with high consumption of refined grains, sugar, and saturated and trans fatty acids may induce inflammation (Giugliano et al., 2006). In a case‐control study, it was shown that dietary pattern with high content of sweeteners, soft drinks, refined grains, and processed meat could increase inflammatory markers (Schulze et al., 2005). The possible reasons for lack of association between unhealthy pattern and abnormal muscle strength could be high consumption of tea and coffee, whose role was reported in other studies in reducing the inflammation (Andersen et al., 2006; Hamer, 2007; Kempf et al., 2010; Steptoe et al., 2007), and reducing the odds ratio of sarcopenia by muscle protection (Guo et al., 2014; Jang et al., 2018; Kim et al., 2013a, 2013b). We also examined the association between dietary patterns and BMI, muscle mass, fat mass and muscle strength by comparing the average level of those in tertiles of each dietary pattern. Compared with those in the lowest tertile of mixed diet, those in highest tertile had lower BMI and muscle mass. This can be due to less energy and protein intake in the highest tertile compared with lowest tertile.

There are several limitations in this study; first, dietary patterns were extracted based on FFQ. FFQ is considered the most appropriate collection tool for dietary data in large epidemiological studies, but estimating the food intake by FFQ might be inaccurate (Hu, 2002; Khani et al., 2004). Second, because this study uses cross‐sectional design, it is not possible to infer causality. Third, the results of analyzing dietary patterns are dependent on the studied population, therefore, one may observe significant differences in dietary pattern according to geographical area, race and culture. Fourth, in the factor analysis, the researcher's theoretical or optional decisions for food grouping and their naming can affect the results (Martinez et al., 1998). Fifth, in this study, dietary pattern was assessed based on participants’ dietary intake and the participants’ dietary behaviors such as pattern, time and number of snack and meals were not investigated.

In conclusion, we found no association between abnormal muscle strength and dietary pattern with combined characteristics of healthy and unhealthy foods among the Iranian middle‐aged men and women. It is suggested to conduct well‐design cohort study to clarify the association of different dietary habits and muscle strength as well as sarcopenia.

## CONFLICT OF INTEREST

The authors have declared no conflict of interest.

## AUTHOR CONTRIBUTION

**Mehnoosh Samadi:** Conceptualization (equal); Investigation (equal); Methodology (equal); Project administration (equal); Writing‐original draft (equal). **Tina Khosravy:** Data curation (equal); Project administration (equal). **Leila Azadbakht:** Methodology (equal); Validation (equal). **Mansour Rezaei:** Formal analysis (equal); Software (equal). **Mohammad Mosafaghadir:** Data curation (equal); Project administration (equal). **Negin Kamari:** Investigation (equal); Project administration (equal); Writing‐original draft (equal). **Amir Bagheri:** Data curation (equal); Investigation (equal); Project administration (equal). **Yahya Pasdar:** Project administration (equal). **Farid Najafi:** Project administration (equal). **Behrouz Hamzeh:** Project administration (equal). **Davood Soleimani:** Validation (equal); Writing‐review & editing (equal).

## ETHICAL APPROVAL

The study protocol was approved by the research ethics committee by the ethics committee at the Kermanshah University of Medical Sciences (Ethic number: IR.KUMS.REC.1396.512). The RaNCD cohort was conducted in accordance with the principles of the Helsinki Declaration. All patients provided written consent for participation in this study.

## Data Availability

The data that support the findings of this study are available on request from the corresponding author. The data are not publicly available due to privacy or ethical restrictions.
